# Carrageenan as a Potential Factor of Inflammatory Bowel Diseases

**DOI:** 10.3390/nu16091367

**Published:** 2024-04-30

**Authors:** Paulina Komisarska, Anan Pinyosinwat, Mutaz Saleem, Małgorzata Szczuko

**Affiliations:** Department of Human Nutrition and Metabolomics, Pomeranian Medical University, 71-460 Szczecin, Polandmutaz.saleem@gmail.com (M.S.)

**Keywords:** carrageenan, inflammatory bowel diseases, Crohn’s disease, ulcerative colitis

## Abstract

Carrageenan is a widely used food additive and is seen as a potential candidate in the pharmaceutical industry. However, there are two faces to carrageenan that allows it to be used positively for therapeutic purposes. Carrageenan can be used to create edible films and for encapsulating drugs, and there is also interest in the use of carrageenan for food printing. Carrageenan is a naturally occurring polysaccharide gum. Depending on the type of carrageenan, it is used in regulating the composition of intestinal microflora, including the increase in the population of Bifidobacterium bacteria. On the other hand, the studies have demonstrated the harmfulness of carrageenan in animal and human models, indicating a direct link between diet and intestinal inflammatory states. Carrageenan changes the intestinal microflora, especially *Akkermansia muciniphilia*, degrades the mucous barrier and breaks down the mucous barrier, causing an inflammatory reaction. It directly affects epithelial cells by activating the pro-inflammatory nuclear factor kappa-light-chain-enhancer of activated B cells (NF-kB) pathway. The mechanism is based on activation of the TLR4 receptor, alterations in macrophage activity, production of proinflammatory cytokines and activation of innate immune pathways. Carrageenan increases the content of Bacteroidetes bacteria, also causing a reduction in the number of short chain fatty acid (SCFA)-producing bacteria. The result is damage to the integrity of the intestinal membrane and reduction of the mucin layer. The group most exposed to the harmful effects of carrageenan are people suffering from intestinal inflammation, including Crohn disease (CD) and ulcerative colitis (UC).

## 1. Introduction–Food Additives Associated with IBD

In recent years, the Western diet has undergone the most significant change, being rich in fats, salt, food additives, sugar, and lacking sufficient fibre. Foods characterized by the aforementioned features are termed ultra-processed foods [1]. Food additives are defined as substances not consumed naturally, the deliberate use of which is intended to yield specific results in food products [2]. They are used to improve colour, taste, texture, smell, nutritional value, and extend the shelf life [3]. These include sweeteners, flavour enhancers, emulsifiers, stabilisers, antioxidants, thickeners, and preservatives [4]. The use of individual food additives must comply with regulations approved by the European Food Safety Authority (EFSA) [5]. The safety of food additives is determined relative to a safe dose for animals, then divided by 100 to obtain a safe quantity for humans [6]. Some of them seem to be associated with inflammatory bowel diseases (IBD), including Crohn’s disease (CD) and ulcerative colitis (UC). The aetiology of IBD is not known, but one of the key elements is the diet and dietary habits [7,8]. In industrialised countries, these diseases show an increasing trend in incidence. Currently, they affect 150–200 people per 100,000 inhabitants [9]. Moreover, individuals with IBD, consume food additives at a higher rate, especially in the paediatric group [10].

### 1.1. Use of Carrageenan and Food Thickeners, Viscosity Modifiers

There has been an alarming increase in the use of carrageenan as an additive in the Western diet in recent years [11]. In the 1980s, carrageenan consumption in the United States ranged from 45–100 mg [12,13]. 

Currently, the highest consumption occurs in the United States, with an average value of 250 mg/day [14]. A significant number of food products contain at least one or more emulsifiers. Individuals with IBD were found to have higher exposure to emulsifiers compared to healthy individuals [15]. Thickeners are substances that swell in water, possess gelling properties, and form sticky, viscous solutions [6]. These include agar, guar gum, carrageenan, xanthan gum, and many others. Attention is drawn to substances used to induce inflammation in animal models [16,17,18,19]. They are not digested by humans, thus directly interacting with the gut microbiota along the entire length of the digestive tract [20,21]. Despite their relative safety, it has been shown that emulsifiers may promote inflammatory bowel diseases in rodents. These include carrageenan and carboxymethylcellulose (CMC) [22,23]. Dextran sulfate sodium salt (DSS) is a substance that acts similarly and is used to induce inflammation in animal and cellular models. In comparison to carrageenan, it activates different inflammatory cytokines, yet the actions and consequences are nearly identical. DSS is not recommended in children’s dietary products, yet it has virtually identical effects to carrageenan. DSS salt leads to the secretion of other inflammatory cytokines such as interleukins (IL-18 and IL-1β), which are dependent on the NLRP1 inflammasome. It also triggers ASC and caspase-1 activation. Abnormal expression or dysregulated function of inflammasomes has been associated with autoimmune diseases [24]. In contrast, carrageenan is associated with cytokines such as IL-6, IL-8, and the TLR4 and BCL10 pathways. DSS is also a less researched food additive due to the lack of certain studies, including those assessing its effects on gastric acid and its influence on pepsin activity. Research on the impact and consequences of consuming emulsifiers is still ongoing. The studied emulsifiers included sodium carboxymethylcellulose (CMC), polysorbate 80 (P80), soy lecithin, sunflower lecithin, maltodextrin, propylene glycol alginate, iota carrageenan, kappa carrageenan, lambda carrageenan, xanthan gum, arabic gum, guar gum, agar agar, diacetyltartaric acid ester of mono- and diglycerides, hydroxypropyl methylcellulose, sorbitan monostearate, mono- and diglycerides, glyceryl stearate, glyceryl oleate. The addition of CMC, P80, maltodextrin, lambda carrageenans, and guar gum to the system resulted in the increased expression of pro-inflammatory molecules from the microbiota [25].

### 1.2. Products Containing Carrageenan

Carrageenan can be found in many food products, mainly in dairy [26]. This is due to carrageenan’s ability to interact with milk proteins. Together with casein, it forms relatively strong macroscopic bonds [27]. When combined with pectin, it exhibits flavour-reinstating properties, which is why this combination is used in light products and skim milk [28,29]. Kappa-type carrageenan forms weak gels in the aqueous phase of milk and is also used in the production of edible films and coatings [30,31]. It is also added to meat products and plant-based beverages such as soy milk [32]. Carrageenan is also used in infant formula preparations [33]. In the future, carrageenan will be used in the pharmaceutical industry to increase viscosity and control drug release, thereby extending the retention of active substances [34]. However, this is a futuristic vision, and numerous research projects are still ongoing to better understand the carrageenan architecture [35].

## 2. Material and Methods

To identify relevant studies, databases such as PubMed, Scopus, and Embase were searched. Inclusion criteria included studies that examined the impact of carrageenan on the body and food additives. Keywords included IBD, carrageenan, inflammatory bowel diseases, Crohn’s disease, ulcerative colitis, food. Studies encompassed both beneficial and adverse effects in animals, humans, as well as animal and human tissues The review included publications concerning non-specific inflammatory bowel diseases in humans were considered. The review included studies evaluating the effects of carrageenan and polysaccharides on IBD in both humans and animals. Studies from the last 20 years were included. Data regarding CGN (E 407) food were considered. Food additives were further coded into subcategories for CGN; κ-, ί- or λ-CGN, unspecified CGN, degraded CGN. The results are presented based on based on a variety of publications including research papers, toxicokinetic publications, and risk assessment opinions. All articles collected in the search process were reviewed based on the abstract. Articles unrelated to the topic and duplicates in the searched databases (PubMed, Embase, Web of Science, Scopus) were excluded.

### Structure of Basic Research

**Figure 1:**
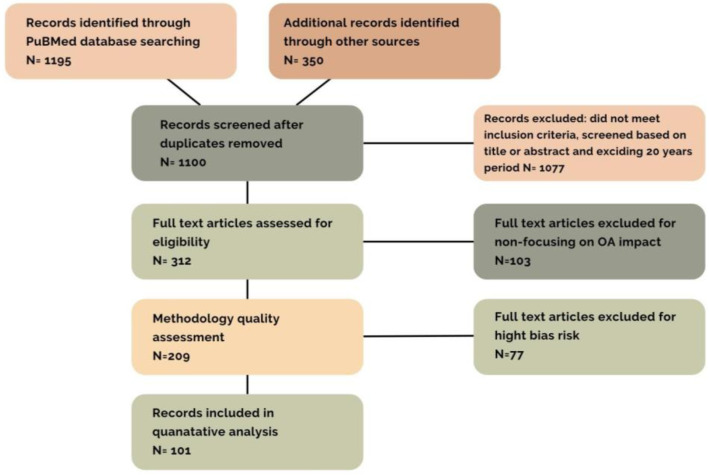
The literature search scheme.

The subject matter was evaluated in accordance with the most recent literature published up to 10 February 2024. The study is based on a systematic analysis of available research and articles (*n* = 1545) focusing on carrageenan, IBD, and carrageenan use. Concepts were linked with nutrition, supplementation, dysbiosis, probiotics, and carrageenan-containing food. To achieve this, international databases such as PubMed, Scopus, Embase were searched, and other available sources from the last 20 years were found. All articles (*n* = 1100) collected in this search process were reviewed. Publications unrelated to the topic, duplicates in databases (PubMed, Scorpus and Embase), conference abstracts, and articles written in languages other than English were excluded (*n* = 101). Only full-text publications concerning carrageenan and inflammatory bowel disease (IBD) were considered (Figure 1). Keyword combinations used in the study included the following: IBD—carrageenan, UC—carrageenan, CD—carrageenan, carrageenan–utilization, carrageenan–health. In case of information duplication in the article, those contributing the most to the topic were utilised.

## 3. Structure of Carrageenan

**Figure 2:**
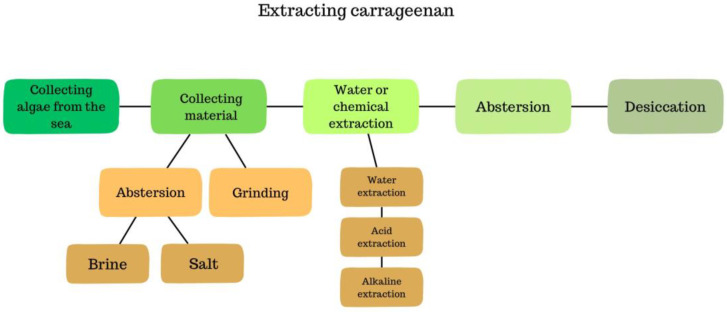
Process of carrageenan extraction.

**Figure 3:**
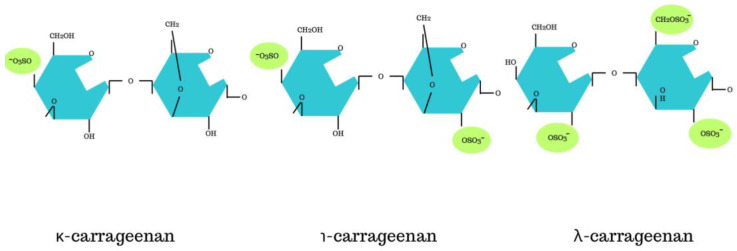
Chemical formulas of individual types of carrageenan.

Carrageenan belongs to the family of naturally occurring polysaccharide gums. It is obtained through the extraction of red seaweeds. Initially, *Chondrus crispus* growing in Ireland, the United States, Canada, and the Iberian Peninsula were used. Currently, other algae found in Japan, Africa, Indonesia, and Chile are also used [28]. Production is based on two species, *Kappaphycus* and *Eucheuma*, which together account for almost 100% of global production [36]. They are harvested from specialized marine farms, most commonly in tropical oceanic regions such as Vietnam, the Philippines, Indonesia, and Tanzania. Algae are cultivated on special marine farms, then harvested and purified. Extraction can occur through water, acidic (e.g., hydrochloric acid), and alkaline (sodium hydroxide) hydrolysis. It is thought that polysaccharides have galactoside characteristics [37]. Carrageenan extraction is presented in Figure 2. Carrageenans are composed of molecules of D-galactose and 3,6-anhydro-D-galactose, linked by β-(1→3)- or β-(1→4)-glycosidic bonds. In the food industry, κ-, ι-, and λ-carrageenan are utilised [37]. It is thought that the use of these three types of carrageenan is related to the presence of one, two, or three sulphated ester groups per repeating disaccharide unit [38]. Figure 3 shows the kappa, iota and lambda structure of carrageenan.

The degree of sulfation is determined based on the 4 and 6 carbon residues in the composition of 3,6-anhydro-D-galactose. It is thought that carrageenan is a polymer chain consisting of repeating disaccharide units, sulphates, and galactose residues, which influence its functionality. Carrageenans are a group of complex polysaccharides that have different types and structures depending on the species. It is thought that carrageenan is a polymer composed of repeating disaccharide units. However, the exact structure may vary depending on its type (kappa, iota, or lambda) and degree of sulfation. They have a molecular weight exceeding 10^5^. It is not possible to have derivatives with a lower mass, as this could have an adverse effect on the human digestive system. Higher molecular weight carrageenans are attributed with protective and even anticarcinogenic effects [39]. Depending on the type of carrageenan, it is used in specific food products. Kappa carrageenan forms strong, firm gels in the presence of potassium ions. Iota carrageenan forms a soft, fluffy structure in the presence of calcium ions. Lambda carrageenan is typically used for thickening dairy products [40]. All carrageenans can be dissolved in hot water (80 °C) as well as in milk at the same temperature. However, it should be noted that not all carrageenans can be dissolved in water at 20 °C. These include iota-carrageenans, lambda carrageenan, and sodium salts of kappa carrageenan. In cold milk, only lambda carrageenan can dissolve [12]. Despite carrageenan’s similarity to fibre, it is not classified into this group. The main reason is that fibre is not subject to digestion in the human digestive system. Carrageenan does not meet this criterion because it does not pass through the body unchanged. It is broken down by gut bacteria and can serve as a source of energy.

### 3.1. Factors Promoting Inflammation through Carrageenan

There are factors that enhance the impact of carrageenan on the body. According to some studies, these include a high-fat diet and a reduction in anti-inflammatory bacteria [41]. When a high-sucrose diet is combined with carrageenan, the length of the colon significantly decreases, and deeper intestinal damage occurs. Conversely, a combination of a high-salt and sucrose diet increases intestinal permeability and significantly reduces goblet cells [42]. It is probable that harmful low-molecular-weight carrageenan can be formed through acid hydrolysis during digestion [33]. Currently, there are no studies presenting genetic predispositions indicating a weaker tolerance to carrageenan consumption. However, an intriguing approach appears seems to be linking the genes of individuals with ulcerative colitis (UC) to a weaker tolerance to carrageenan [43].

### 3.2. Mechanism of Carrageenan Action

#### 3.2.1. Animal Model

Animal models underwent comprehensive analysis. Due to the lack of digestibility in the earlier parts of the gastrointestinal tract, carrageenan directly interacts with the gut microbiota and can be utilized by gut microorganisms. Studies have demonstrated alterations in the composition of the gut microbiome, resulting in a reduction in the thickness of the intestinal barrier and mucin content [24]. Mucin is a glycoprotein that forms the intestinal mucus layer [44]. Upon the removal of carrageenan from the diet, wound healing occurred, and inflammatory reactions decreased [23,45]. In a mouse model, the use of lambda carrageenan demonstrated a dependence on Toll-like receptor 4 (TLR4) and MyD88 activation of innate immunity. Studies indicate clear activation of innate immunity by lambda–carrageenan, which is dependent on the TLR4. The induction of pro-inflammatory cytokines like tumour necrosis factor (TNF-α) and IL-6 occurred, which showed abnormal activity in macrophages. Furthermore, it was demonstrated that tolerance levels and the maintenance of intestinal homeostasis are significant factors in the context of oral carrageenan administration [46]. Another model showed the effect of kappa carrageenan on existing intestinal inflammation in mice. The use of carrageenan increased mortality and caused greater weight loss. One of the reasons was the increased expression of TLR4, IL-6, TNF-α and nuclear factor kappa-light-chain-enhancer of activated B cells (NF-κB) in the colonic mucosa [47]. Further research indicates that kappa carrageenan enhances the inflammatory response in vitro and in vivo by increasing the quantity of the TLR4 receptor. Carrageenan binds to TLR4, thereby blocking the binding of lipopolysaccharide (LPS), a component of Gram-negative bacteria. It also mediates increased LPS expression and the secretion of IL-8 and TNF-α directly by LPS. When cells are exposed to pathogens, carrageenan exacerbates the inflammatory response. It is responsible for increasing the amount of inflammatory cytokines [48]. In a porcine model, the administration of a 1% carrageenan solution resulted in gut dysbiosis and initiation of inflammation in the colon. This model is analogous to the human model, and it has also been demonstrated that the reaction to carrageenan may not occur in healthy individuals without infection or prior inflammation [49]. Furthermore, kappa carrageenan also caused a decrease in short-chain fatty acids (SCFA). A number of the aforementioned animal models posit that carrageenan is a contributing factor in the exacerbation of existing gastrointestinal inflammation [50].

#### 3.2.2. Human Model

**Figure 4:**
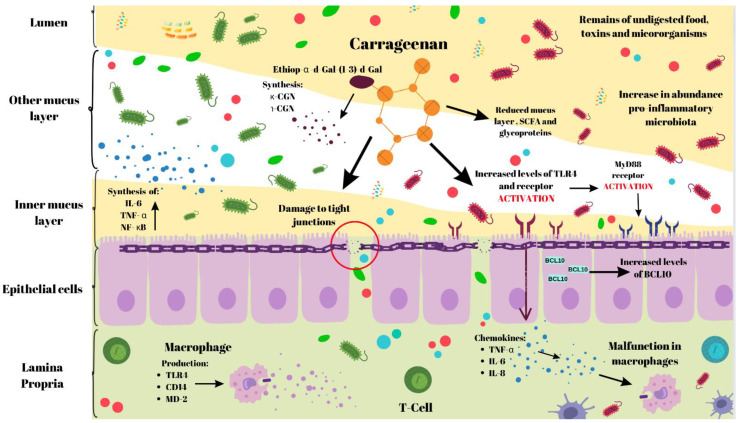
The carrageenan-induced inflammation model [11]. TNF-α—tumour necrosis factor α; NF-κB—the nuclear Factor kappa B; IL-6—interleukin 6; IL-8—interleukin 8; SCFA—short chain fatty acids; BCL10—BCL10 gene; TLR4—Toll-like receptor 4; created using BioRender.com, https://app.biorender.com/, accessed on 22 April 2024.

Cultivated human epithelial cells of the mucous membrane react to carrageenan by inducing inflammation in the colon. The specific κ-CGNases or ι-CGNases cause an increase in IL-8 and immune signalling adaptor BCL10 concentrations. The reaction occurs through the α-d-Gal-(1–3)-d-Gal epitope on carrageenin and TLR4 [51]. Another study demonstrated that exposure to carrageenan resulted in increased expression of Bcl10, as well as cytoplasmic and nuclear NF-κB. Furthermore, activation of the IL-8 promoter was observed. This led to the initiation of a separate inflammatory pathway, which is similar to that occurring in NOD2 mutation, previously known as CARD15. This pathway is a genetic predisposition to Crohn’s disease. This may constitute compelling evidence linking genetic and environmental aetiology of IBD [52]. Further evidence indicates that lipopolysaccharide LPS activation in Gram-negative bacteria, leads to Toll-like receptor (TLR) activation in intestinal epithelial cells. Bcl10 serves as the mediator of induction. IL-8 activation occurs in human intestinal epithelial cells (IEC) [53]. Additionally, it has been demonstrated that degraded carrageenan negatively affects macrophage cells. This results in increased TLR4, CD14, and MD-2 expression, as well as enhanced NF-κB pathway activity [54]. Monolayered intestinal epithelium cocultured with THP-1 cells (a monocyte isolated from peripheral blood from an acute monocytic leukemia patient) is similarly susceptible to damage by carrageenan, a polysaccharide found in red algae. This damage is accompanied by an increase in the secretion of IL-6, IL-1β, and TNF-α [55]. In conclusion, it has been demonstrated that carrageenan triggers innate immune inflammatory pathways, where TLR4 and BCL10 play crucial roles [56,57]. A randomised, cross-over controlled trial compared short-term use of high-molecular-weight carrageenan in patients with ulcerative colitis. The study did not find evidence that short-term carrageenan use exacerbates ulcerative colitis [58]. Another study administered carrageenan to patients with ulcerative colitis in remission. Two groups were studied, one consuming capsules containing carrageenan, and the other had placebo capsules. The results indicated that carrageenan intake contributed to earlier disease relapse in remission-phase individuals, up to 50% of cases [59]. Furthermore, carrageenan was found to increase MIC-1 expression, which is a macrophage-inhibiting cytokine in human enterocytes. This promotes apoptosis of epithelial cells [60]. A few studies have shown that carrageenan may interfere with digestive proteolysis by disrupting gastric juice secretion, which may increase the risk of malnutrition [61,62]. In our opinion, this requires further extensive investigation and confirmation of the mechanism. The carrageenan-induced inflammation model is shown in Figure 4.

## 4. Gut Dysbiosis

Dysbiosis refers to an imbalance in the gut microbiota, which includes all microorganisms inhabiting the gastrointestinal tract, including bacteria, fungi, eukaryotes, and viruses [63,64]. The gut microbiota plays an essential in digestion, absorption, metabolism, and immune shaping [65]. Prolonged alteration of the gut microbiota composition may be one of the causes of IBD [66]. The consumption of ultra-processed food creates an environment conducive to the proliferation of microorganisms associated with inflammatory states [67]. Another factor contributing to dysbiosis is increased proteolytic activity [68]. *Bacteroides* and *Pseudomonas* may influence the induction of inflammation in the colon [69]. There are numerous predispositions to dysbiosis. Loss of the anti-inflammatory bacterium *Akkermansia muciniphila* may be significantly relevant to exacerbating carrageenan-induced colonic inflammation [70]. Studies have also confirmed the promotion of pro-inflammatory *Prevotella* bacteria by kappa carrageenan [71]. Moreover, it has been demonstrated that the development of inflammation may vary depending on the bacterial flora composition of the individual [51]. The most significant factor contributing to dysbiosis is primarily diet, although other factors such as stress, food additives, antibiotics, and the overuse of non-steroidal anti-inflammatory drugs (NSAIDs) also play a role [72]. Some of these factors can be eliminated relatively quickly, while others require more effort, such as relaxation techniques or effective stress management [73].

## 5. Associated Symptoms after Carrageenan Consumption

Some of the most commonly reported symptoms after carrageenan consumption include rectal bleeding, weight loss, ulcers, and diarrhoea [74]. In most cases, there is also a disruption of the integrity of the intestinal mucosa. Mucosal integrity is controlled by tight junctions and adherens junctions to the epithelium [75]. Additionally, there is also an infiltration of pro-inflammatory cytokines [76]. Another typical symptom is the overproduction of reactive oxygen species by leukocytes and macrophages, which plays a significant role in carrageenan-induced inflammation [77].

### 5.1. Alleviation Process of Symptoms

The mechanism of carrageenan action mainly relies on damaging the intestinal mucosa. One method involves treating with liposaccharide (LPS), which alleviates damage to the cell membrane integrity [78]. Studies have shown a reduction in oxidative stress and anti-inflammatory properties of fucoidan extracts from *Macrocystis pyrifera* [79]. The use of certain bacteria can also help alleviate symptoms, including Bast A and Bact B, derived from strawberries (BACT1), and bananas (Bact 2) [80]. Probiotic therapy with strains such as *Lactobacillus* and *Bifidobacteria* exerts antioxidant and anti-inflammatory effects [81]. Recent research indicates that A. muciniphila has a positive impact on the composition of gut microbiota metabolic functions through the PI3K/Akt pathway [82]. Administration of D-methionine and butyric acid may have protective and anti-inflammatory effects [83]. In some cases, faecal transplant has resulted in the reversal of symptoms induced by carrageenan [50].

### 5.2. The Therapeutic Potential of Marine Polysaccharides

The intestines can be the site of entry for various infections, are in contact with food allergens, and are home to a vast number of bacteria, whose composition needs to be carefully managed [84]. The basic division of seaweeds is based on their colour: red, green, and brown [85,86]. The main components of brown seaweeds are alginates and fucoidan, whose effects have been clinically proven [87]. They exhibit low toxicity, biodegradability, and high compatibility, indicating their safety as food ingredients [88]. The main active ingredient in green algae is Ulvan, a water-soluble polysaccharide [89]. It has been demonstrated to possess anti-hyperlipidaemic, anti-inflammatory, antiviral, antioxidant, antithrombotic, and immunomodulatory properties [90,91,92]. In contrast to other seaweeds, red seaweeds contain bioactive ingredients. The three main components of red seaweeds are carrageenan, xylan, and agar [93,94,95]. Carrageenan is one of the most well-studied polysaccharides in terms of nutritional safety and toxicity [96,97]. In addition to the aforementioned adverse effects, carrageenans, contingent on their molecular weight, may exhibit anti-inflammatory, antioxidant, and immunomodulatory effects [98].

## 6. New Findings

### 6.1. Carrageenan in Various Dietary Patterns

The widespread use of carrageenan raises concerns regarding colon health. The state of the intestines can be influenced by the food we consume. Western diets, which are rich in simple carbohydrates, fats, sugar, salt, food additives, and lacking in fibre, have undergone radical changes. Western dietary patterns have been categorised into three categorised: a diet rich in sucrose, a diet rich in salt and sucrose, and a diet rich in salt. Carrageenan has been included in all these diets, and in all cases, intestinal health was observed to deteriorate. The diet rich in salt and sucrose caused more severe damage to goblet cells, significantly reducing their quantity, and increasing intestinal permeability. The sucrose-rich diet was characterized by shortened intestines and damaged crypts. In all cases, intestinal inflammation was observed to worsen. Current dietary habits significantly exceed the recommended amount of sugar and salt, which may increase the risk of inflammation. It is important to note that, high-molecular-weight carrageenan was used in the study, which is commonly used in the food industry worldwide [42]. Carrageenan, has been shown to alter gut microbiota, particularly *A. muciniphilia*, degrades the mucosal barrier, provoking an inflammatory response. It directly affects epithelial cells by activating the NF-kB pro-inflammatory pathway. Experimental studies on cells (in vitro) and in most cases on animals indicate an enhanced inflammatory response, altering the mucus layer and gut microbiota composition. All these factors contribute to the secretion of inflammatory factors such as cytokines and activation of innate immune pathways. It seems that carrageenan affects humans through two pathways: activating the NF-kB pathway and altering the microbiota composition, reducing beneficial bacteria while increasing pro-inflammatory bacteria [99].

### 6.2. The Impact of Carrageenan and the Gut Microbiota

**Figure 5:**
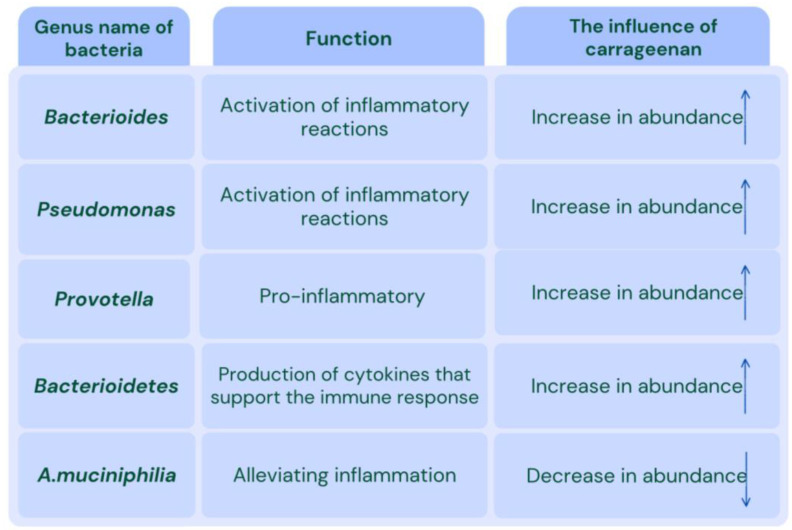
The effect of carrageenan in the intestinal microbiota.

Some studies have reported a positive effect of carrageenan on human health, while the majority of studies have demonstrated negative effects when carrageenan is introduced into the diet or treatment process. The introduction of high-molecular-weight carrageenan as a treatment supplement has been observed to exacerbate inflammation, even with short-term treatment. A THP-1 reaction has been observed, which destroyed Caco-2. The study considered the effect of carrageenan on the intestinal mucosal structure and bacterial metabolism. A 90-day treatment period led to a 13% increase in intestinal load. Groups receiving carrageenan exhibited a significant increase in the content of Bacteroidetes bacteria, with an average increase of up to 54%. In contrast, the amount of *Akkermansia muciniphilia* decreased by an average of 70%, while the content of Proteobacteria decreased by 4.6%. The number of SCFA-producing bacteria decreased, with their average content in faeces dropping by 63%. The utilisation of kappa carrageenan has been observed to enhance the consumption of nutrients from mucus by gut bacteria, exceeding SCFA production. This phenomenon has been linked to the disruption of intestinal integrity [50]. The administration of carrageenan has been demonstrated to induce an increase in the number of genes encoding polysaccharide-binding proteins and enzymes, which in turn facilitates the degradation of mucin. This process has been observed to result in a reduction of the mucus layer by an average of 59.5%. Notably, in germ-free models, the mucus layer has been found to remain unaltered. Bacteria demonstrate the ability to penetrate mucous membranes in the presence of carrageenan. It is possible that dysbiosis of microorganisms may be a key factor influencing susceptibility to carrageenan-induced inflammation [50]. The effect of the carrageenan in the intestinal microbiota are shown in Figure 5.

### 6.3. Probiotic Therapy and Postbiotic Therapy to Alleviate Carrageenan Effects

**Figure 6:**
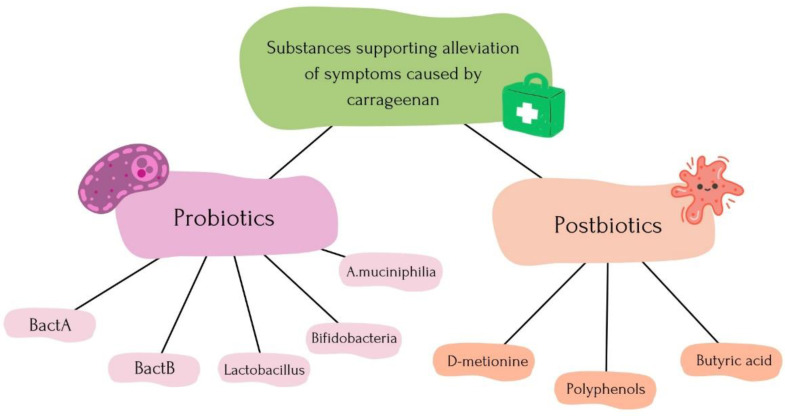
Substances alleviating the impact of carrageenan on the gastrointestinal system.

Probiotic intervention during inflammation has been shown to increase SCFA content, restore the intestinal mucosal layer and thereby regain its thickness by 56%. SCFA production contributes to maintaining immunological homeostasis and strengthening tight junctions. Submucosal oedema has also been shown to decrease. Work is underway on new types of bacteria that could alleviate intestinal inflammation and its consequences. These include BactA, isolated from strawberries, and Bact B, isolated from bananas. They have demonstrated symptom relief and the ability to suppress the disease. Bact A may stimulate the population of anti-inflammatory bacteria such as *Akkermansia muciniphilia*. They also regulate interleukin-1, which is a kinase in the signalling pathway mediated by the Toll-like receptor, which has become the subject of new research in inflammatory disease treatment. It is also important to note that certain bacteria can exacerbate inflammation, including *Bacteroides* and *Pseudomonas*. During active disease processes, it is crucial to monitor the quantity of bacteria that support the suppression of inflammation, such as *Lactobacillus* and *Bifidobacteria* [80,81]. *Akkermansia muciniphilia* is of particular interest as it not only affects the state of the intestines but also acts on the entire gut-liver axis. It reduces the levels of IL-6 and IL-10, decreases neutrophil and macrophage infiltration, strengthens the intestinal barrier by inhibiting LPS, regulates gut dysbiosis, restores SCFA production by improving levels of acetic acid, butyric acid, and 2-methylbutyric acid. The increase in the amount of *A. muciniphilia* also results in an increase in the amount of *Lactobacillus* bacteria [82]. In addition to probiotics themselves, the use of postbiotics, which contain non-living organism components including SCFAs, peptides, enzymes, and proteins, appears beneficial. Recent studies indicate a reduction in weight loss through supplementation with butyrate and D-methionine. They reduce the expression of inflammatory genes. Furthermore, butyric acid has been demonstrated to possess mild anti-inflammatory effects through the activation of the NRF2 signalling pathway. NRF2 has been shown to possess antioxidant and anti-inflammatory properties. During the inflammatory process, its expression is reduced, but butyrate and D-methionine have been shown to increase its expression. Butyrate has been shown to inhibit the growth of pathogenic bacteria while increasing the growth of beneficial bacteria [84]. The substances that alleviate the impact of carrageenan on the gastrointestinal system are shown in Figure 6.

### 6.4. Carrageenan Use among People

The short-term consumption of high-molecular-weight carrageenan by people with UC did not lead to disease activation. However, the study group consisted of only seven people who consumed carrageenan in the form of cocktails for seven days. Further research with a larger study group and longer consumption time is necessary to determine the safety of carrageenan for humans [58]. It is also important to note that randomised studies have demonstrated that individuals with IBD, including those with UC and CD, consume a greater quantity of food containing additives in the form of emulsifiers.

### 6.5. New Applications of Marine Algae including Carrageenan

High-molecular-weight carrageenan with a mass of 200 kDa–800 kDa can be utilised in new branches of industry, both in the food and technological sectors. Carrageenan can be employed in the creation of edible films and for encapsulating drugs. Additionally, the production of plant-based meat products represents a novel concept. Nevertheless, the most significant interest is aroused by the utilisation of carrageenan for 3D/4D printing, where it can be employed as a wall material, edible items, and food ink.

Due to the increasing frequency and intensity of adverse reactions to some synthetic drugs, new solutions are sought among natural products. One such example is marine algae which possesses antithrombotic, hypoglycaemic, and antioxidant properties. In the case of autoimmune diseases, immunosuppressive drugs are used. Polysaccharides derived from algae such as alginate, fucoidan, ascophyllan, and porphyran when applied in the form of nanoparticles can provide new solutions in the treatment of inflammatory diseases [87]. Extracts from seaweeds are rich in metabolites such as proteins, vitamins, polyunsaturated fatty acids, and antioxidants. These components can be extracted, mainly for the commercial industry. Polysaccharides from red algae can act as prebiotics and effectively regulate the composition of gut microbiota, as some of our bacteria demonstrate the ability to digest fibre from these seaweeds. The use of carrageenan derived from Kappaphycus algae has been shown to increase the population of Bifidobacterium bacteria, which is an interesting application [96]. However, low-molecular-weight carrageenan is considered a food contaminant, and there is also insufficient research on carrageenan proteolysis to determine its degradation and its impact on human health [100].

## 7. Conclusions

In recent years, there has been a growing interest in the relationship between dietary behaviours, dysbiosis, and the composition of gut microbiota. The inclusion of carrageenan in the diet in studies has resulted in a decrease in the quantity of anti-inflammatory bacteria, especially *A. muciniphilia*, and exacerbated the growth of pro-inflammatory microbiota. Additionally, the mucin layer decreased, leading to intestinal barrier dysfunction in many cases. This directly affected epithelial cells through the activation of the pro-inflammatory NF-kB pathway. No studies have been conducted indicating genetic predispositions and reduced tolerance to carrageenan.

Research on humans still provokes controversy and reluctance. However, a few conducted studies have shown the activation of ulcerative colitis (UC) and shortened remission time after high molecular weight carrageenan supplementation.

A significant majority of studies indicate negative effects carrageenan. The mechanism is based on the activation of the TLR4 receptor, alterations in macrophage activity, production of proinflammatory cytokines and activation of innate immune pathways. Similarities have been shown between the effects of carrageenan and changes in the NOD2/CARD15 gene, which are predisposing factors for Crohn’s disease via LPS activation of Gram-negative bacteria. The most vulnerable group to the harmful effects of carrageenan is individuals with IBD.
